# Insights into the functional biology of schistosomes

**DOI:** 10.1186/1756-3305-4-203

**Published:** 2011-10-20

**Authors:** Anthony John Walker

**Affiliations:** 1 School of Life Sciences, Kingston University, Kingston upon Thames, Surrey, KT1 2EE, UK

## Abstract

The need to discover new treatments for human schistosomiasis has been an important driver for molecular research on schistosomes, a major breakthrough being the publication of the *Schistosoma mansoni *and *Schistosoma japonicum *genomes in 2009. This 'Primer' considers recent advances in the understanding of schistosome biology by providing a snapshot of selected areas of contemporary functional schistosome research, including that on the genome, the tegument, cell signalling and developmental biology, offering biologists a valuable insight into the life of these fascinating parasites at the basic and molecular level.

## What are schistosomes?

**Figure 1:**
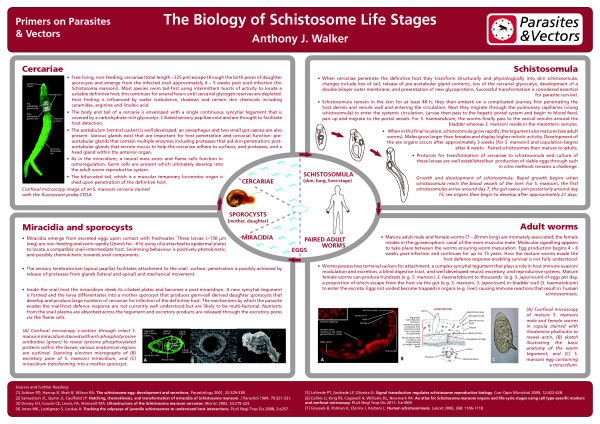
**The biology of schistosome life stages**.

Schistosomes (phylum: Platyhelminthes) are blood-dwelling parasites that mature as separate-sex adults in the veins of mammals and birds. Throughout their complex life-cycle, these trematodes undergo striking morphological and physiological changes with individual life-stages displaying distinct adaptations both to parasitic life, and also to free-living life that permits movement between definitive-vertebrate and intermediate-snail hosts. Such adaptations include cilia or tails for swimming, secretory glands for host penetration, a tegument and glycocalyx for parasite protection/host immuno-modulation, a gynaecophoric canal for sustained pairing between sexes, muscular suckers for attachment/feeding, and highly organised reproductive systems for efficient fertilization and egg production (Figure 1, [1-7]; for a downloadable version of the poster see Additional File 1). Research on schistosomes continues to largely centre on three species of schistosome that infect humans (*Schistosoma mansoni, Schistosoma haematobium, and Schistosoma japonicum*) and are the focus of this 'Primer', with most studies having been performed on *S. mansoni*.

## Why study the biology of schistosomes?

Like many other parasites with complex life-cycles, schistosomes are fascinating organisms to study, particularly in the context of developmental biology and host-parasite relationships. Not only has co-evolution resulted in intricate interplay between the parasite and its snail or vertebrate host but also between the adult male and female worms. Much recent research on the basic biology of schistosomes has been driven strategically by the need better to control human schistosomiasis, with the identification of new drug targets [8,9] and vaccine development [9,10] being key determinants. This is not without good reason; schistosomiasis is estimated to affect over 200 million people in 76 developing countries [7,11], with over 700 million people at risk, and the currently-available drug, praziquantel, has been used in mono-therapy for several decades, so wide-spread emergence of drug resistance is possible. Pathology associated with human schistosomiasis is not due directly to the adult worms but rather the large numbers of eggs that become trapped in tissues during egg migration, or after embolism in organs such as the liver, spleen or lungs. The fibrotic granulomas that form around the eggs develop as a consequence of a strong CD4+ Th2 response that is regulated by various cell types, cytokines and chemokines with certain reactions limited by CD4+ regulatory T-cells [12]. Given the importance of the eggs to disease progression and transmission, schistosome development, pairing, sexual maturation and egg production remain active areas of fundamental research. In this 'Primer', selected areas of contemporary functional research on schistosomes are introduced by exploring three advances made in the last decade; three topics of schistosome biology that are ripe for investigation are then considered.

## Schistosome biology: three advances in the last decade

### The genome

Complementing previous transcriptomic work [e.g. [13]], the eagerly-awaited *S. mansoni *and *S. japonicum *genomes were published in July 2009 [14,15]; together, these have provided an invaluable resource for the schistosome research community, although genome studies on *S. haematobium *are badly needed [16]. Analyses of the existing genomes and predicted proteomes have revealed a wealth of information highlighting mechanisms by which the parasite might exploit host nutrients and cell signalling molecules to support growth, and revealing the nature of schistosome neuropeptides, kinases, ion-channels, metabolic pathways, and proteolytic enzymes required for host invasion and haemoglobin degradation [14,15]. The availability of sequence data has since supported research projects spanning a wide spectrum of schistosome biology from studies into miracidial cilia beat [17], regulation of spermatogenesis and oogenesis [18], gender-specific gene expression [19], and histamine signalling [20] in adult worms, through to vaccine discovery using immunomics [10] and characterization of genes encoding small RNA regulatory pathway components [21]. In addition, a detailed functional annotation of the *S. mansoni *kinome comprising 252 eukaryotic protein kinases [22] has recently been enabled, paving the way for further research into schistosome kinases; such research is particularly pertinent to research on schistosome development and survival and modulation of schistosome cell signalling pathways by host molecules. *In silico *approaches, reliant upon genome data, have also recently been employed to prioritize potential *S. mansoni *drug targets [23]. Thus, through supporting basic research that integrates experimental and bioinformatic approaches, it is anticipated that the genome will help deliver novel anti-schistosome drug and/or vaccine candidates. In addition, the availability of the schistosome genome and predicted proteome will also bring huge benefit to research in comparative biology and the evolution of parasitism, with the future assembly of the genome of the free-living flatworm *Schmidtea mediterranea *[24] being particularly important to this endeavour.

### The molecular nature of the adult worm tegument

The tegument of schistosomula and adult worms is intriguing. It includes a single multinucleated cytoplasmic layer (syncytium) that covers the entire worm and is linked to underlying nucleated cell bodies by cytoplasmic connections that span the musculature (reviewed in [25]; see poster). The apical surface of the tegument undergoes dynamic turnover and has a unique architecture comprising two closely aligned lipid bilayers, the plasma membrane and the host-proximal membranocalyx. This surface plays a vital function in immune evasion/modulation and nutrient uptake thus ensuring schistosome survival; in addition, it has recently been proposed that the tegument functions in the removal of waste lactate from adult schistosomes [26]. During the last decade, advances in proteomic/genomic technologies have enabled studies into the identification of proteins present in the tegument of adult schistosomes. The rationale for much of this work is that tegument proteins might represent useful drug/vaccine targets given their close proximity to host blood. In 2006, proteomic analysis of *S. mansoni *proteins obtained by differential solubilization of an apical membrane preparation identified 51proteins based on homology with known proteins in other organisms [27]. Among these were enolase involved in energy metabolism; the molecular chaperone heat shock proteins 19, 17 and 20, calmodulin; various cytoskeletal and molecular motor proteins including actin, severin and dynein light chains; mitochondrial proteins such as ATP synthase; vesicle proteins, and plasma membrane transporters; enzymes and structural molecules such as calcium ATPase, glucose transport protein, alkaline phosphatase, annexin and tetraspanins A, B, and C [27]. This study advanced knowledge from that achieved a year earlier which identified 43 tegument proteins that included those possibly present within the syncitium [28]. In 2010, by first biotinylating proteins on the surface of live adult *S. japonicum *and subsequent capture using streptavidin beads, 54 proteins were identified by tandem mass spectrometry (MS/MS), the majority of which are putatively surface-exposed [29]. Comparative analysis of the results obtained with those of other studies revealed that many of these identified proteins are commonly expressed in both *S. mansoni *and *S. japonicum *[29]. More recently, by employing trypsin to release the most accessible surface proteins from live *S. mansoni*, analysis by MS/MS revealed the presence of host complement proteins C3 and C4, the leukocyte marker CD44 and various schistosome proteins including annexins IV, V and VI, the membrane protease calpain, and Sm200 and Sm25, proteins of unknown function [30]. In addition, release of GPI-anchored proteins using phosphatidylinositol-specific phospholipase C revealed the presence of schistosome Sm29, CD59a and b, Sm200, carbonic anhydrase, alkaline phosphatase, and ADP-ribosyl cyclase [30]. These studies highlight the power of proteomics and subsequent data mining in identifying important molecules present at the host-parasite interface and the ingenious approaches used by researchers to obtain relevant fractions for study. Other proteins were found during these proteomic studies that lack homology to proteins expressed in other organisms including humans; these unique proteins are therefore ripe for further investigation as in addition to being potential therapeutic targets understanding their function might yield valuable insight into the specific nature of schistosome-host interactions.

### Cell signalling and development of the schistosome reproductive system

Schistosomes possess separate sexes. An interesting feature of schistosome conjugal biology is that sustained pairing occurs between males and females. Molecular signalling between them is essential for complete development of the female reproductive apparatus including the ovary and vitellaria, and separation of worm couples reverses this maturation process. Between 2001 and 2007 strong evidence emerged for the transforming growth factor β (TGFβ) signalling pathway playing an important part in female reproductive development and egg embryogenesis; this pathway involves TGFβ growth factors that activate serine/threonine kinase transmembrane receptors (TβRI/TβRII) which in turn signal to downstream elements of the Smad pathway [reviewed in [31,32]]. Both TGFβ and bone morphogenic protein (BMP) subfamily members have been discovered in schistosomes with *S. mansoni *BMP characterized recently [33]. Importantly, RNA interference-mediated knockdown of the TGFβ superfamily member Inhibin/Activin in eggs aborts their development highlighting a crucial role for this molecule in schistosome embryogenesis [34]. In addition, other studies highlighted that Src, Src/Fyn and Syk cytoplasmic tyrosine kinases probably govern reproductive development in a distinct manner with SmTK3/SmTK5 expressed in the ovary, vitellaria and testes, and SmTK4/SmTK6 in ovary and testes but not in the vitellaria [32]. Interestingly, SmTK3 was recently found to interact with the formin-homology protein SmDia which also binds the small GTPase SmRho1 in male and female worm gonads; given the role of such components in other organisms it is considered that these co-operative pathways might organize reproductive cytoskeletal events [35]. A polo-like kinase, SmPlk1, has also been found to play a major role in *S. mansoni *reproduction; not only were SmPlK1 transcripts detected in the female vitelline cells and oocytes and in male spermatocytes, but a novel Plk1 inhibitor disrupted the gonads resulting in defective oogenesis and spermatogenesis [36]. Finally, in *S. japonicum*, a Frizzled member (SjFz9) representing a novel receptor of the evolutionarily conserved Wnt developmental signalling pathway has been found to be predominantly expressed in the testes of male worms and the ovary and vitellaria of the female worm [37], providing tantalizing opportunities for investigating the role of this protein in the development of these tissues. Taken together, these findings demonstrate some of the excellent progress made to decipher factors governing reproductive development of schistosomes and thus egg production. Such work will undoubtedly influence the direction of schistosome reproductive research within the coming decade, providing a springboard for the development of effective drugs that target key proteins involved in egg production by adult worms.

## Schistosome biology - three areas ripe for research

### Regulation of schistosome development

Our overall knowledge of the molecular control of schistosome development remains poor. The large morphological and physiological differences that exist between each of the schistosome life-stages means that identification of important drivers of development, particularly those that govern key life-stage transitions, will be a major task. Such work needs to be considered in the context of changing environments, both within a host and between hosts, and the different metabolic milieux present. For example, the importance of human and snail growth factors to development of the requisite life-stages need to be evaluated and the mechanisms by which host-derived molecular signals are communicated to the parasite to benefit this process understood. Despite such challenges, we do have some insight into the changes in gene expression that occur during schistosome development [38,39] and of potential regulators of reproductive development (above). In addition, knowledge gleaned from studies into the regulation of development of early post-embryonic snail-host life-stages [40,41] could inform similar research on definitive-host stages and vice-versa. As with other organisms, molecular regulation of schistosome development will be complex, but studies this area are important to develop a complete understanding of schistosome developmental biology, crucial for drug development work, and to inform research in comparative developmental biology including that on other trematode parasites such as *Fasciola *spp.

### Schistosome sensory systems

What are the cellular mechanisms used by schistosomes to sense the presence of the intermediate and definitive hosts? What mechanisms enable them to respond to environmental cues and migrate within their hosts to their sites of final development? What mechanisms enable immature adult worms to recognize each other, couple, and sustain their intimate association? All of these fascinating questions relating to schistosome sensory biology remain ripe for investigation. Sensory structures exist on the surface of adult schistosomes and on various regions (e.g. the terebratorium) of certain larval stages, but how signals received at the parasite surface modulate the behaviour of the parasite remains largely unknown. There has however been excellent progress in understanding various aspects of the schistosome neuronal system, particularly in relation to the presence of neurotransmitters [42,43] and some G-protein coupled receptors [20,43]. Molecular communication between such components will be vital to behavioural responses such as schistosome muscle contraction and motility of schistosome larvae, but how such signals are integrated to provide a co-ordinated response remains to be explored. Although this represents a substantial research challenge, understanding the molecular control of schistosome behaviour is crucial to developing a detailed knowledge of schistosome functional biology.

### The variant nature of a schistosome within its host

While parasites such as *Plasmodium falciparum *and *Trypanosoma brucei *are well known to use polymorphism and protein variation to evade host immune responses [44,45], the extent to which a schistosome varies its molecular appearance *via *genetic mechanisms to facilitate survival in its host is little understood. It has however recently been shown that, in the snail host, *S. mansoni *produces mucins coded by a multi-gene family whose members frequently recombine; multiple splice variants also exist for each gene and as a consequence mucin polymorphism occurs [46]. Moreover in *S. japonicum*, the tegument protein tetraspanin-2 has been found to be diverse with sequence variation occurring on the surface of the molecule [47], and more recently a mechanism of protein variation generated by differential splicing of micro-exon gene transcripts has been elucidated in *S. mansoni *[48]. Understanding the nature of protein variation and polymorphism in schistosomes has major implications for the development of vaccines against these parasites and studies such as these pave the way for novel investigations into schistosome immune evasion strategies.

## Conclusion

'Primers on Parasites & Vectors' are concise articles with restricted coverage and therefore many excellent works published on the topics introduced remain un-cited here. A simple ISI Web of Knowledge search for articles including "*Schistosoma *or schistosome or schistosomiasis" in the title only, reveals 5101 separate research articles published between Jan 2001 and May 2011, indicative of the interest during the last decade in schistosomes and the diseases that they cause. Within the schistosome research community there is a relentless desire better to understand the biology of these parasites and their interactions with their vertebrate and mollusc hosts. Technological advances such as improved success with transient gene silencing in schistosomes by RNA interference [49] have been instrumental in the quest to define function for schistosome gene products. It is anticipated that in the coming decade, emerging technologies and integration of ideas will push forward the boundaries of research focusing on the biology of schistosomes and that fascinating paradigms for schistosome function will emerge.

## Competing interests

The authors declare that they have no competing interests.

## Supplementary Material

Additional file 1 **Downloadable poster describing the biology of schistosome life stages**. Click here for file
