# Supplementary material for: Insights into the functional biology of schistosomes
Source: Parasit Vectors. 2011 Oct 20;4:203. doi: 10.1186/1756-3305-4-203 (PMC3206467; doi:10.1186/1756-3305-4-203)
Supplement: Additional file 1 — Downloadable poster describing the biology of schistosome life stages. [file 1756-3305-4-203-S1.PDF]

The Biology of Schistosome Life Stages

Primers on Parasites & Vectors

Anthony J. Walker

Parasites & Vectors

Cercariae

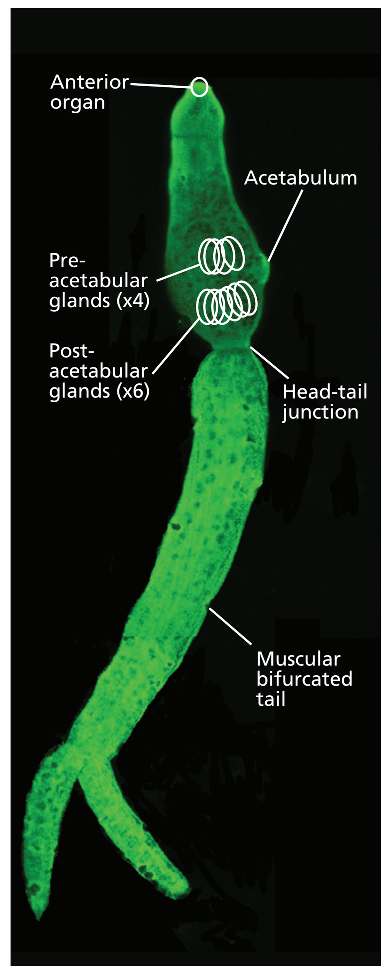

- Free-living, non-feeding, cercariae (total length ~325 µm) escape through the birth pores of daughter sporocysts and emerge from the infected snail approximately 4 – 5 weeks post snail-infection (for *Schistosoma mansoni*). Most species swim tail-first using intermittent bursts of activity to locate a suitable definitive host; this continues for several hours until cercarial glycogen reserves are depleted. Host finding is influenced by water turbulence, shadows and certain skin chemicals including ceramides, arginine and linoleic acid.
- The body and tail of a cercaria is enveloped with a single continuous syncytial tegument that is covered by a carbohydrate-rich glycocalyx. Ciliated sensory papillae exist and are thought to facilitate host detection.
- The acetabulum (ventral sucker) is well developed; an oesophagus and two small gut caecea are also present. Various glands exist that are important for host penetration and cercarial function: pre-acetabular glands that contain multiple enzymes including proteases that aid skin penetration; post-acetabular glands that secrete mucus to help the cercariae adhere to surfaces, and proteases; and a head gland within the anterior organ.
- As in the miracidium, a neural mass exists and flame cells function in osmoregulation. Germ cells are present which ultimately develop into the adult worm reproductive system.
- The bifurcated tail, which is a muscular temporary locomotor organ is shed upon penetration of the definitive host.

Confocal microscopy image of an S. mansoni *cercaria stained with the fluorescent probe CFDA.*

Schistosomula

- When cercariae penetrate the definitive host they transform structurally and physiologically into skin schistosomula; changes include loss of tail, release of pre-acetabular gland contents, loss of the cercarial glycocalyx, development of a double bilayer outer membrane, and presentation of new glycoproteins. Successful transformation is considered essential for parasite survival.
- Schistosomula remain in the skin for at least 48 h; they then embark on a complicated journey first penetrating the host dermis and venule wall and entering the circulation. Next they migrate through the pulmonary capillaries (=lung schistosomula) to enter the systemic circulation. Larvae then pass to the hepatic portal system and begin to blood feed, pair up and migrate to the portal vessels. For *S. haematobium*, the worms finally pass to the vesical venules around the bladder whereas *S. mansoni* reside in the mesenteric venules.

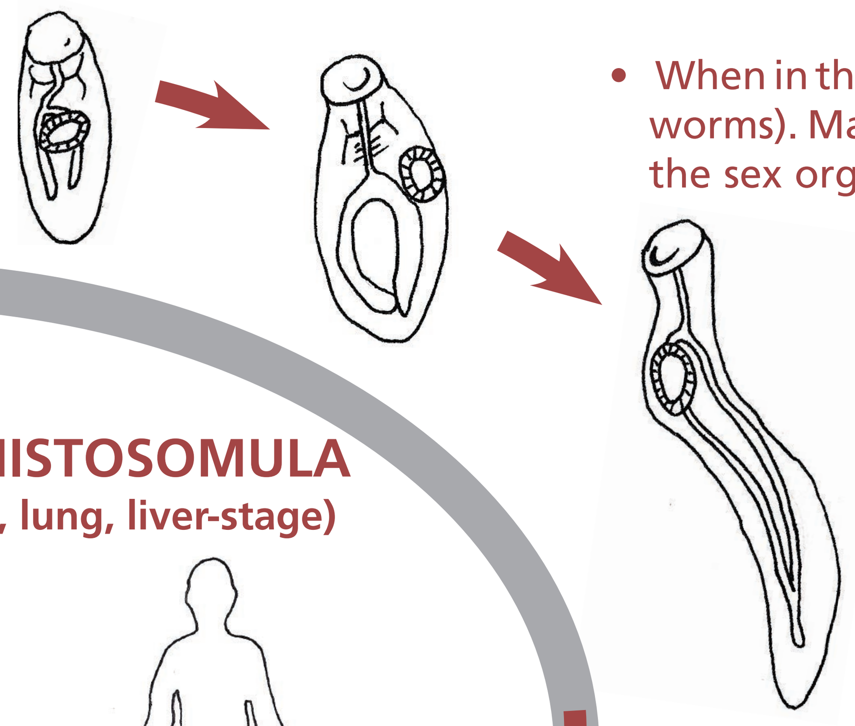

- When in this final location, schistosomula grow rapidly; the tegument also matures (see adult worms). Males grow larger than females and display higher mitotic activity. Development of the sex organs occurs after approximately 3 weeks (for *S. mansoni*) and copulation begins after 4 weeks. Paired schistosomes then mature to adults.
- Protocols for transformation of cercariae to schistosomula and culture of these larvae are well established but production of viable eggs through such *in vitro* methods remains a challenge.

Growth and development of schistosomula. Rapid growth begins when schistosomula reach the blood vessels of the liver. For S. mansoni, *the first schistosomules arrive around day 7; the gut caeca join posteriorly around day 15; sex organs then begin to develop after approximately 21 days.*

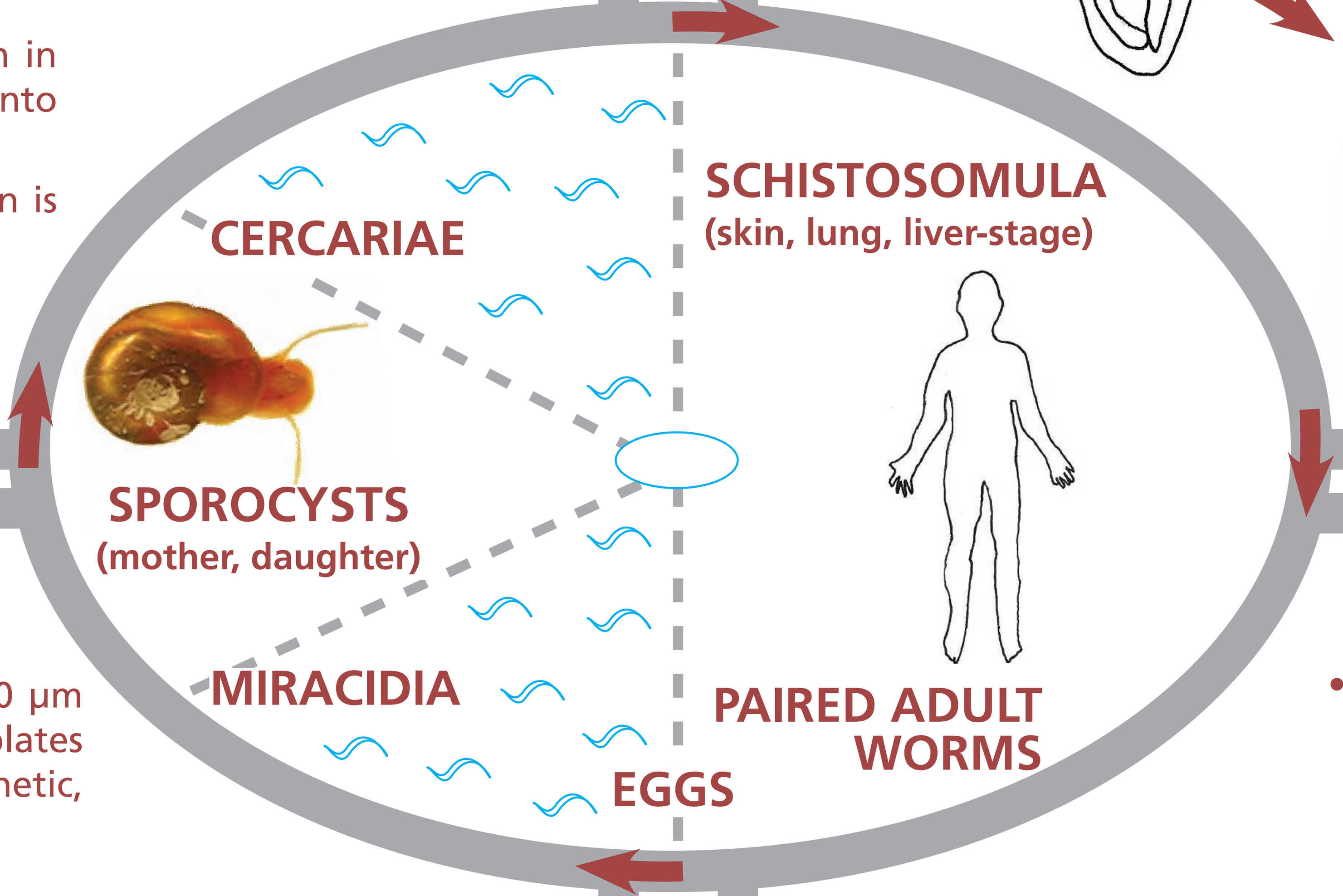

Miracidia and sporocysts

- Miracidia emerge from excreted eggs upon contact with freshwater. These larvae (~150 µm long) are non-feeding and swim rapidly (2mm/s for ~6 h) using cilia attached to epidermal plates to locate a compatible snail-intermediate host. Swimming behaviour is positively photokinetic, and possibly chemokinetic towards snail components.
- The sensory terebratorium (apical papilla) facilitates attachment to the snail surface; penetration is possibly achieved by release of proteases from glands (lateral and apical) and mechanical movement.
- Inside the snail host the miracidium sheds its ciliated plates and becomes a post-miracidium. A new syncytial tegument is formed and the larva differentiates into a mother sporocyst that produces germ-cell derived daughter sporocysts that develop and produce large numbers of cercariae for infection of the definitive host. The mechanisms by which the parasite evades the snail-host defence response are not currently well understood but are likely to be multi-factorial. Nutrients from the snail plasma are absorbed across the tegument and excretory products are released through the excretory pores *via* the flame cells.

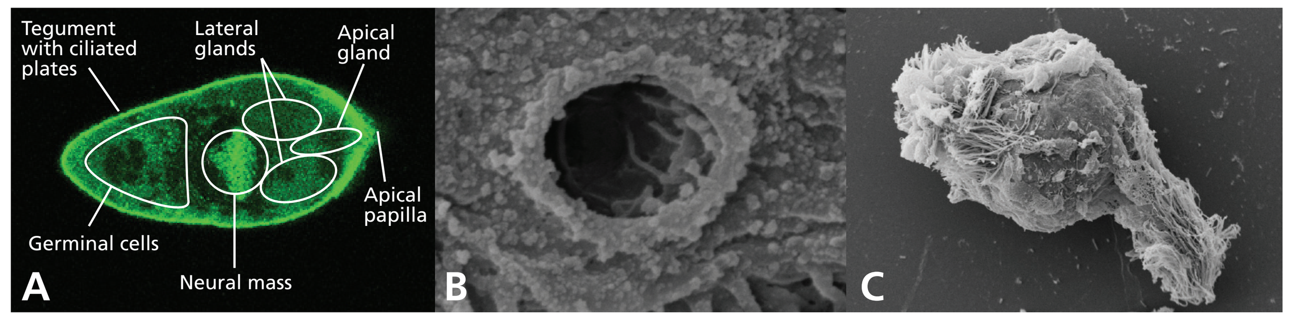

(A) Confocal microscopy z-section through intact S. mansoni *miracidium stained with anti-phosphotyrosine antibodies (green) to reveal tyrosine phosphorylated proteins within the larvae; various anatomical regions are outlined. Scanning electron micrographs of (B) excretory pore of* S. mansoni *miracidium, and (C) miracidium transforming into a mother sporocyst.*

Adult worms

- Mature adult male and female worms (7 – 20 mm long) are intimately associated; the female resides in the gynaecophoric canal of the more muscular male. Molecular signalling appears to take place between the worms ensuring worm maturation. Egg production begins 4 – 6 weeks post-infection and continues for up to 15 years. How the mature worms evade the host defence response enabling survival is not fully understood.
- Worms possess two terminal suckers for attachment, a complex syncytial tegument that plays a role in host immune evasion/modulation and excretion, a blind digestive tract, and well developed neural, excretory, and reproductive systems. Mature female worms can produce hundreds (e.g. *S. mansoni, S. haematobium*) to thousands (e.g. *S. japonicum*) of eggs per day, a proportion of which escape from the host *via* the gut (e.g. *S. mansoni, S. japonicum*) or bladder wall (*S. haematobium*) to enter the excreta. Eggs not voided become trapped in organs (e.g. liver) causing immune reactions that result in human schistosomiasis.

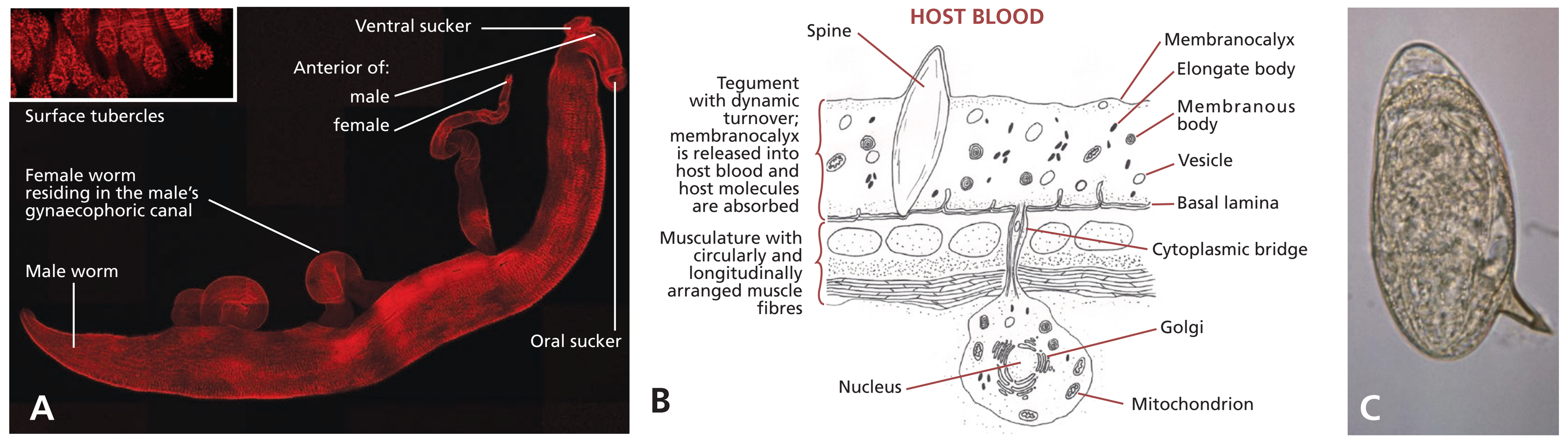

(A) Confocal microscopy of mature S. mansoni *male and female worms in copula stained with rhodamine phalloidin to reveal actin, (B) sketch illustrating the basic anatomy of the worm tegument, and (C)* S. mansoni *egg containing a miracidium.*

Sources and Further Reading:

[1] Ashton PD, Harrop R, Shah B, Wilson RA: **The schistosome egg: development and secretions.** Parasitology 2001, 22:329-338.

[2] Samuelson JC, Quinn JJ, Caulfield JP: **Hatching, chemokinesis, and transformation of miracidia of Schistosoma mansoni.** J Parasitol 1984, 70:321-331.

[3] Dorsey CH, Cousin CE, Lewis, FA, Stirewalt MA: **Ultrastructure of the Schistosoma mansoni cercariae.** Micron 2002, 33:279-323.

[4] Jones MK, Lustigman S, Loukas A: **Tracking the odysseys of juvenile schistosomes to understand host interactions.** PLoS Negl Trop Dis 2008, 2:e257.

[5] LoVerde PT, Andrade LF, Oliveira G: **Signal transduction regulates schistosome reproductive biology.** Curr Opin Microbiol 2009, 12:422-428.

[6] Collins JJ, King RS, Cogswell A, Williams DL, Newmark PA: **An atlas for *Schistosoma mansoni* organs and life-cycle stages using cell type-specific markers and confocal microscopy.** PLoS Negl Trop Dis 2011: 5:e1009.

[7] Gryseels B, Polman K, Clerinx J, Kestens L: **Human schistosomiasis.** Lancet 2006, 368: 1106-1118.
